# Supplementary material for: Shotgun transcriptome, spatial omics, and isothermal profiling of SARS-CoV-2 infection reveals unique host responses, viral diversification, and drug interactions
Source: Nat Commun. 2021 Mar 12;12:1660. doi: 10.1038/s41467-021-21361-7 (PMC7954844; doi:10.1038/s41467-021-21361-7)
Supplement: Supplementary file 1 — Supplementary Information [file 41467_2021_21361_MOESM1_ESM.pdf]

Supplementary Figures

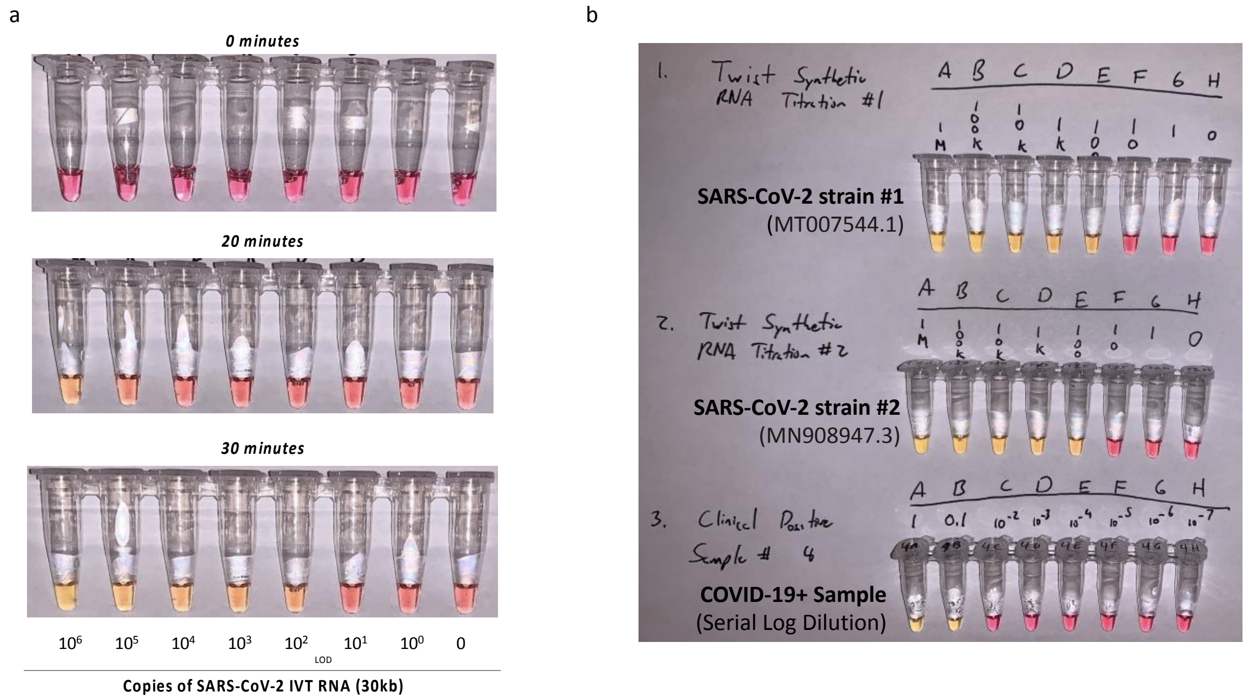

Supplementary Figure 1. Additional Testing and Titration of the LAMP Assay with Synthetic and Clinical Samples. Samples were prepared using the LAMP protocol with a reaction time of 30 minutes. Reaction progress was measured (a) from 0, 20, and 30 minutes. (b) This was repeated for both Twist SARS-CoV-2 synthetic RNAs (MT007544.1, top and MN908947.3, middle*)* from 1 million molecules of virus (10^6), then titrated down by log10 dilutions. Limit of Detection (LOD) range is shown with a gradient after 30 minutes between 10 and 100 viral copies. (bottom) A clinically positive sample that was not detectible by Qubit (<0.05 ng/mL) was nonetheless detected by LAMP, in accordance with detection of low viral titer samples.

Supp. Figure 2

a

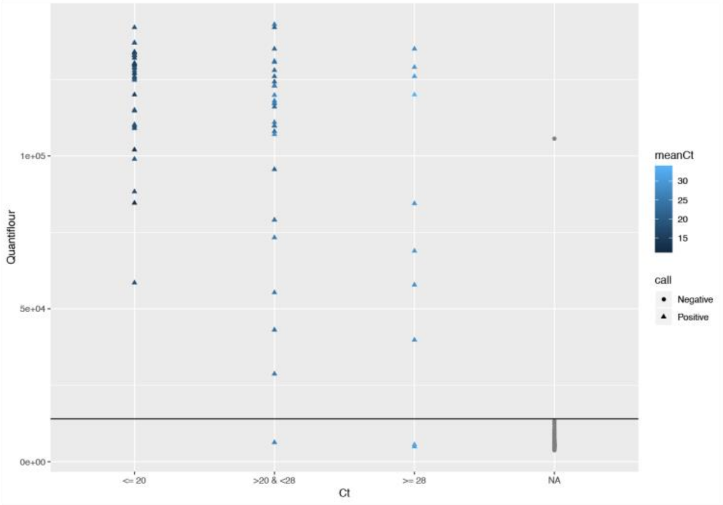

b

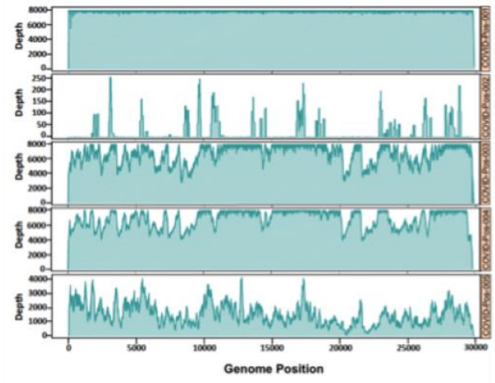

c

Sample	LAMP	CT value	Hybrid Capture	Total # of Reads	# of SARS CoV-2 Reads	# of Human Reads	% Depth of Coverage SARS CoV-2	# of Variants	Estimated Viral Titer (copies/ μl)
COVID-Pos-001	+	23.170	+	24515074	21894339	1768076	100	6	303.3
			+	9504425	8535867	683178	>99.9	6	296.8
COVID-Pos-002	-	*Undetermined*	+	390744	3870	385013	46.4	4	4.8
			-	99445	-	98106	18.6	-	-
COVID-Pos-003	+	24.517	+	2215260	1630169	431570	>99.9	8	210.6
			+	658057	488194	133252	99.95	8	189.9
COVID-Pos-004	+	23.581	+	2150448	1623921	461493	>99.9	11	337.9
			+	1677685	624583	1009045	>99.9	12	314.2
COVID-Pos-005	+	28.327	+	637319	307872	251931	99.94	6	125.0
			+	539371	135703	251931	99.77	7	86.2
COVID-Neg-001	-	*Undetermined*	-	435254	-	435115	-	-	-
			-	1509564	-	1509300	-	-	-

Supplementary Figure 2. Ct and RFU (Relative Fluorescence Units) Thresholds for the LAMP Assay. (a) Assay performance is dependent upon viral load, as measured by qRT-PCR. LAMP assay shows a decrease in sensitivity, reflected as Relative Fluorescence Units measured on the QuantiFluor (Y-axis), as Ct values increase (binned X-axis, NA are samples with undetermined Ct values). Samples positive by qRT-PCR are triangles, negative samples by qRT-PCR are circles. Horizontal line represents ideal quantifluor cut-off. (b) Hybrid capture NGS yielded high coverage across the viral genome. Coverage plots across the viral genome are shown for one replicate for the 5 positive clinical samples with genome position on bottom, depth of coverage on left, sample name on right. (C) concordance across testing modalities.

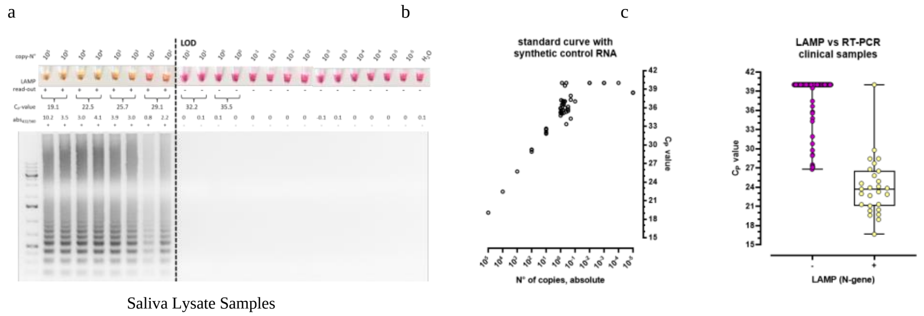

Supplementary Figure 3. Reproducibility, sensitivity, and specificity for the LAMP Assay. (a) Whole oropharyngeal swab lysates from clinical positive (Cp-value >0) and negative samples (Cp = NA) were used to test the LAMP reaction. (b) The standard curve with synthetic RNA was also tested relative to absolute number of copies (x-axis) and the Cp value (y-axis). (c) The Cp value for the LAMP positive (+, right) and negative (-, left) were compared to the Cp value from qRT-PCR (y-axis).

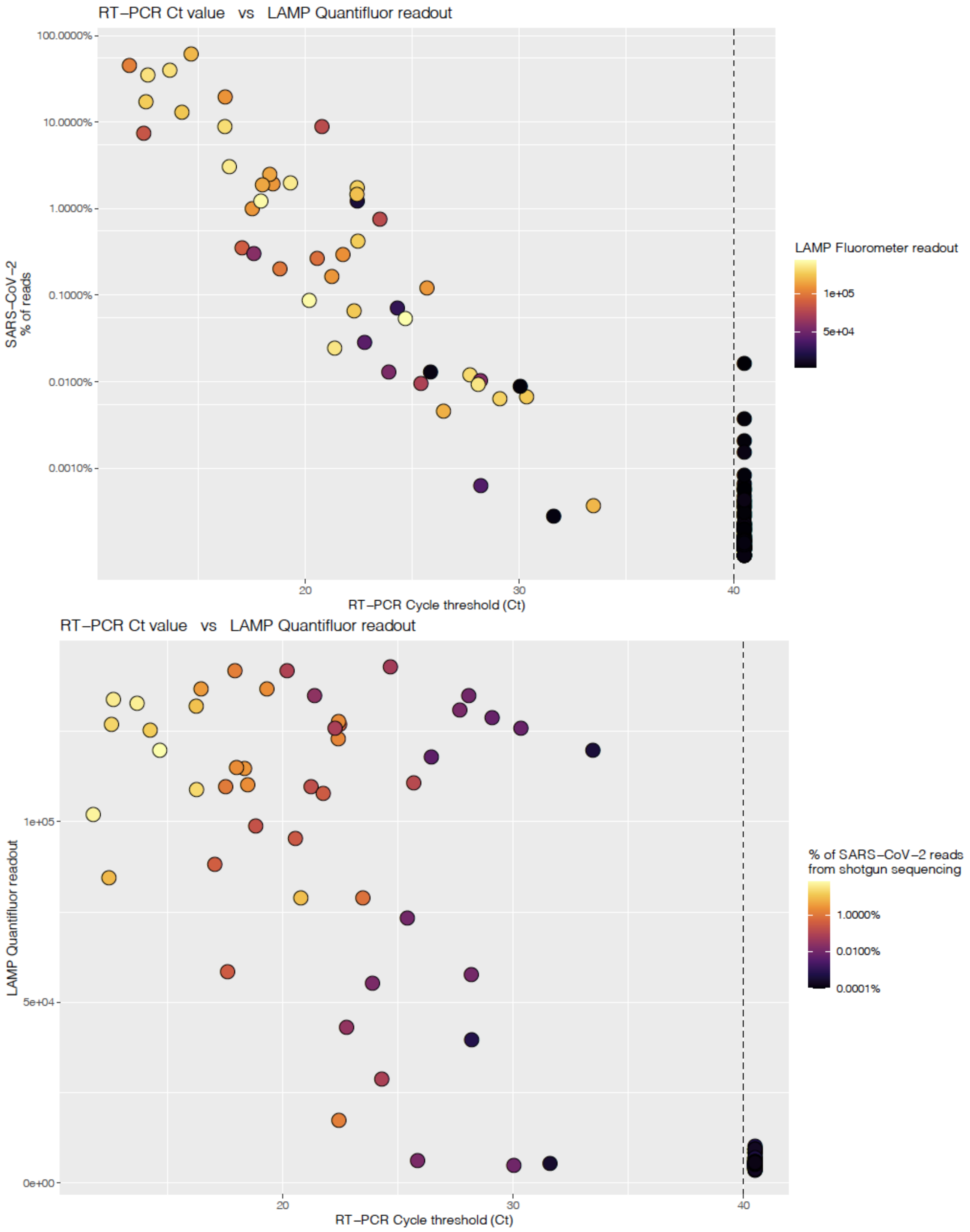

Supplementary Figure 4. (a) SARS-CoV-2 abundance, as measured with NGS and percentage of reads (y-axis) is compared to the Ct Threshold for qRT-PCR (x-axis), with lower Ct values representing higher viral abundance, and the LAMP reaction output (Fluorimeter values, black to yellow scale). Pearson R^2=-0.88. (b) Similar plot as (a), but now comparing SARS-CoV-2 abundance measured by LAMP output (y-axis) and the RT-PCR Cycle (Ct) threshold (x-axis). Pearson R^2=-0.74. Note: This figure includes samples from the protocol optimization and primer development process that do not reflect the final improved specificity and sensitivity of the NEB E2019S LAMP kit.

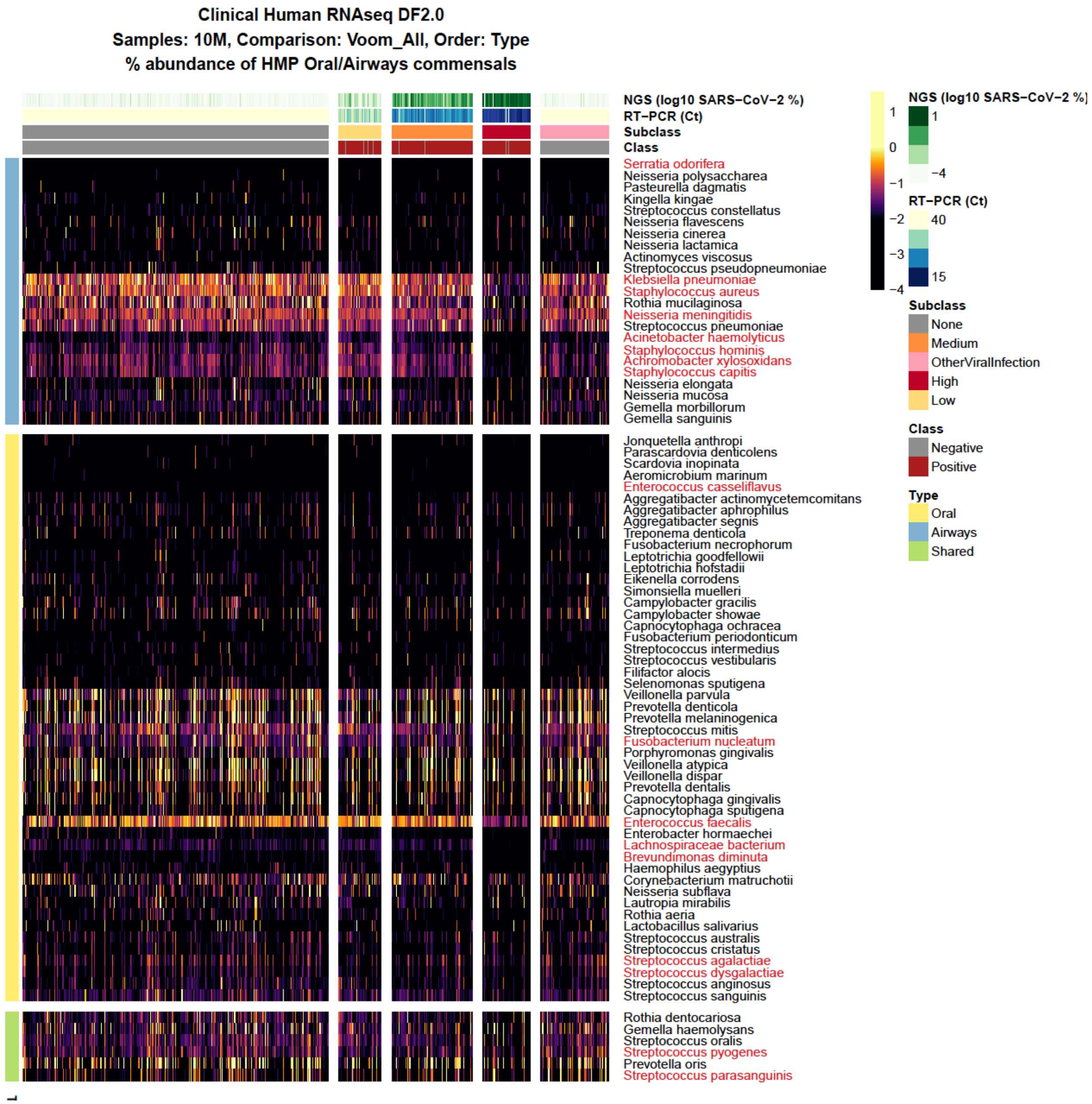

Supplementary Figure 5. Metatranscriptome profiles of the patient cohorts. Common respiratory species plotted as a $\log_{10}$ abundance of mapped reads, with each organism as a line and each vertical column as a patient, with bacteria annotated from the Human Microbiome Project (HMP) as normal airway (blue, top portion), oral (yellow, middle set), or both oral and airway flora (green, bottom). Species with significant differences between the COVID+ and COVID- patients are highlighted in red.

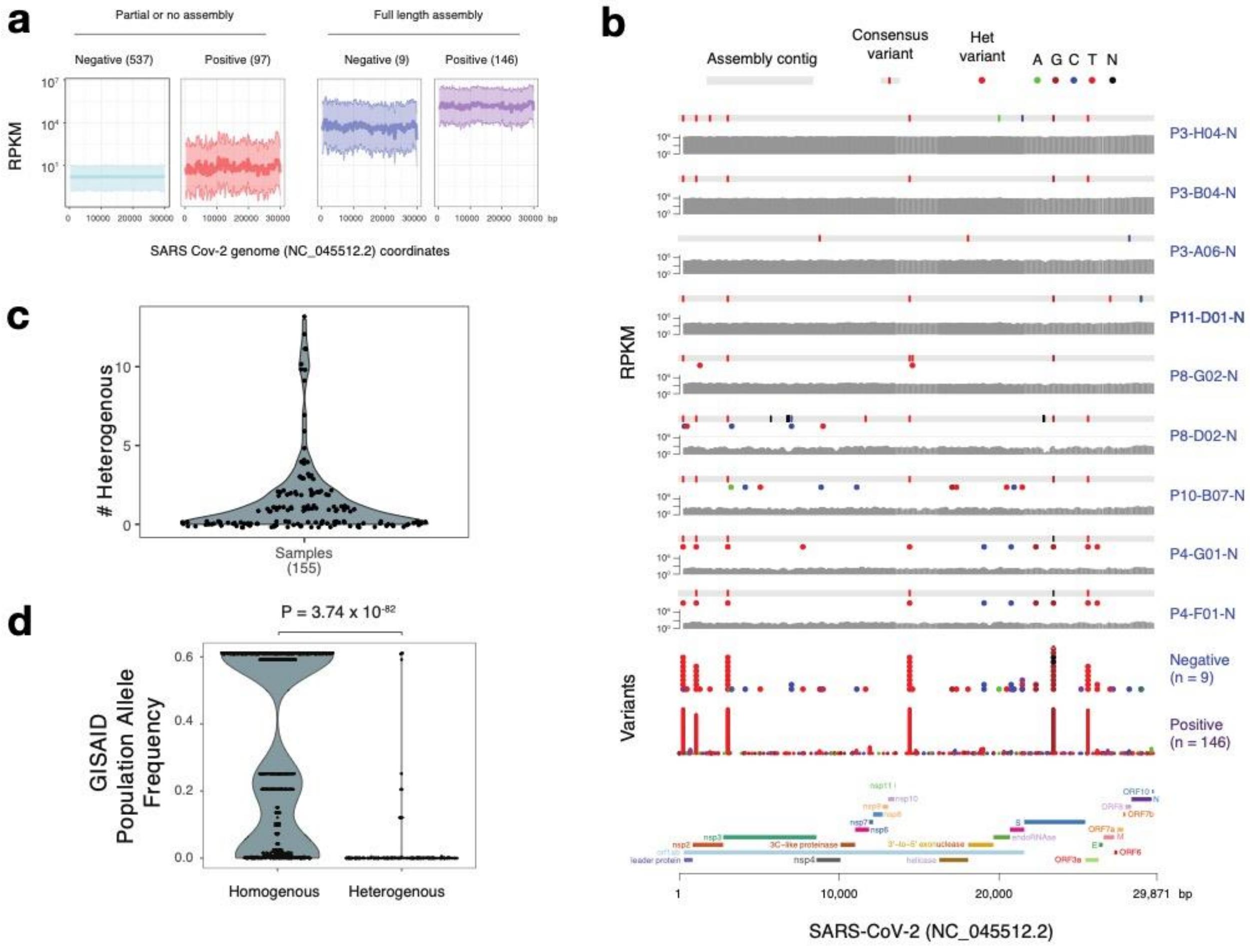

Supplementary Figure 6. SARS-CoV-2 assemblies. (a) Reads per kilo base per million mapped reads (RPKM) of qRT-PCR positive and negative samples across samples from which we recovered a full length assembly of the SARS-CoV-2 genomes (right) and those from which only a partial or no assembly was recovered (left). B. variants across 9 qRT-PCR negative samples from which a full length assembly was recovered (top 9 tracks), summary of all variants in negative and positive samples (bottom two tracks). The positions of genes in the SARS-CoV-2 genome are shown at the bottom of the panel. (c) Violin plot of the total number of heterogenous positions in which of the 155 assemblies. (d) comparison of the population allele frequency between heterogenous and homogenous positions across GISAID samples.

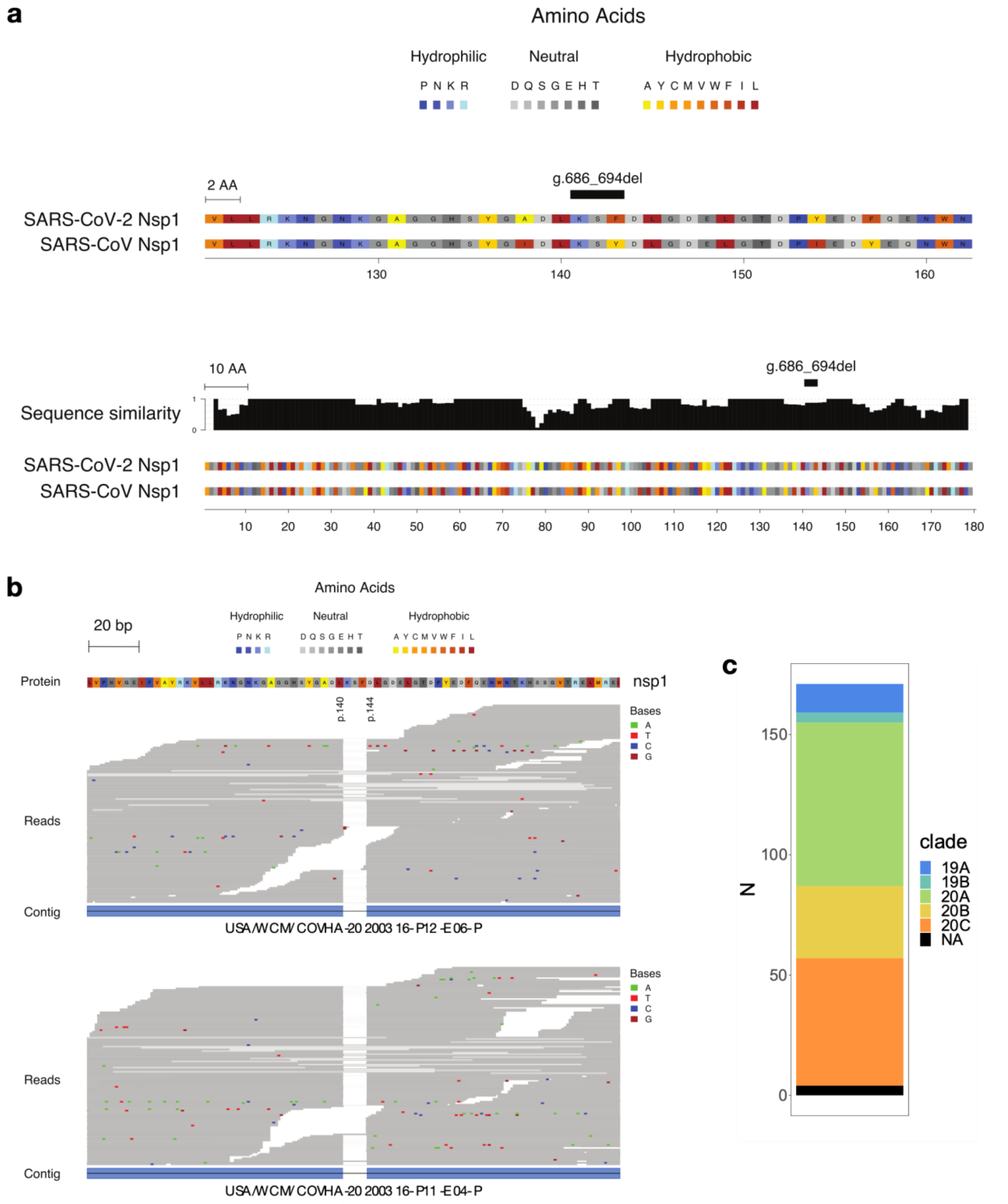

Supplementary Figure 7. Deletion of three amino acids in the SARS-CoV-2 protein Nsp1. (a) alignment of the amino acid sequences of the Nsp1 proteins of SARS-CoV and SARS-CoV-2 (bottom). The top of the panel shows a zoom-in on the region of the deletion. Black bars above the alignment mark the sequence similarity between the two. Amino acids are colored by amino acid type. (b) Read coverage around the deletion in two selected samples. (c) Clade affiliations of GISAID genomes with the Nsp1 deletion (p.141_143del).

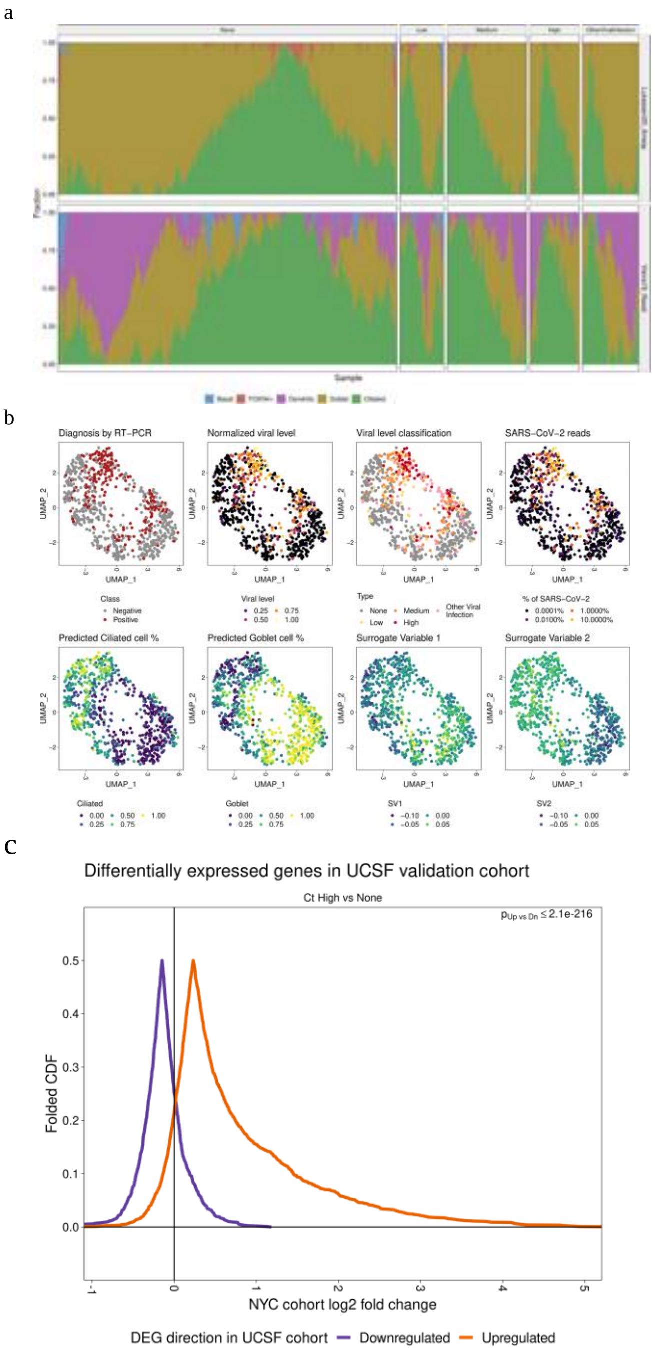

Supplementary Figure 8. Cellular sub-type deconvolution from nasopharyngeal (NP) swabs. The MUSIC algorithm was used to separate the cellular sub-type gene expression signatures present in the total RNA-seq data from the NP swabs. (a) The fraction of cells estimated for each cell type (y-axis) was calculated for the clinical samples that tested positive by RT-qPCR in different viral levels, with proportions shown for goblet cells (yellow), ciliated cells (green), basal cells (blue), dendritic cells (purple) and FOXN4+ cells (red). First row shows deconvolution using the single cell transcription reference set of upper airway cells by Lukassen *et al* 2020. and the second row shows a similar approach using nasal epithelial cells by Vieria *et al*. (b) Samples are embedded in two-dimensions using UMAP dimensionality reduction and separate features of samples are projected onto the points including RT-PCR based diagnosis, viral levels, predicted cell fractions from cell deconvolution. (c) Comparison to the UCSF COVID cohort for differentially-expressed genes, with the upregulated genes (orange) plotted relative to our own here and the down-regulated genes (purple) also showing the same trend.

Supp. Figure 9

a

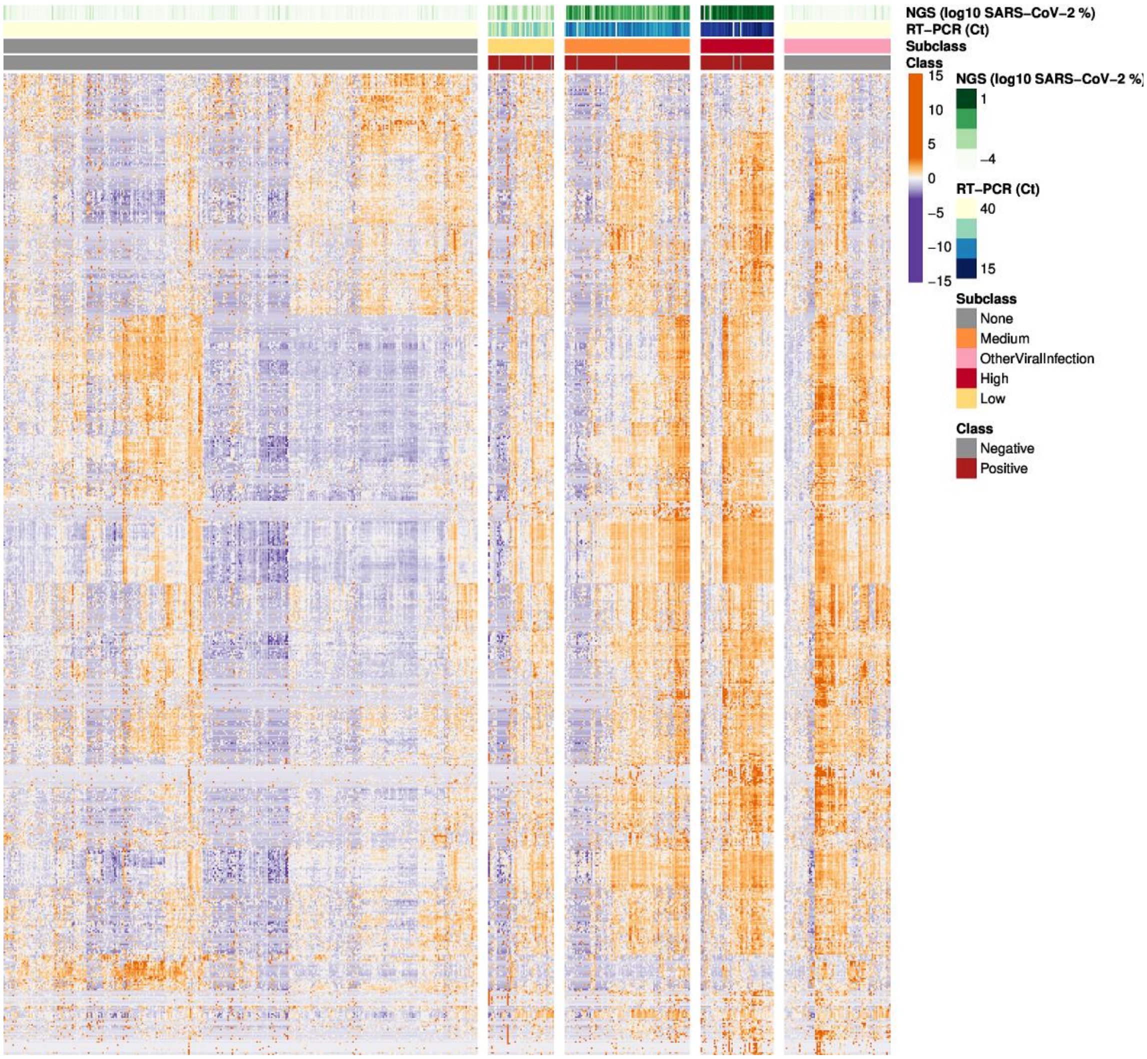

b

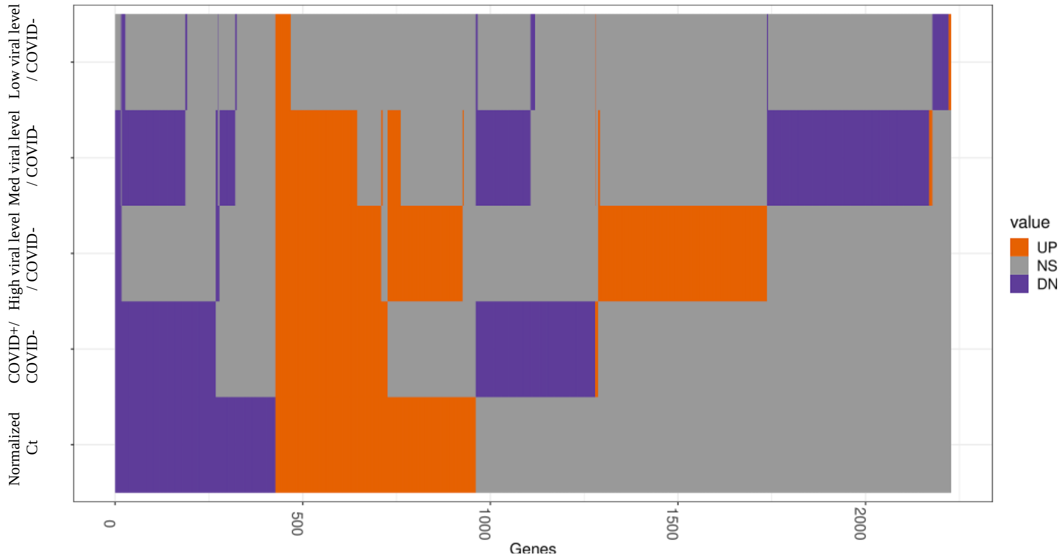

Supplementary Figure 9. Differentially expressed genes.(a) Top rows: samples were quantified by a range of viral detection methods, including LAMP (QuantiFluor), RNA-seq (log10 SARS-CoV-2 % of reads), and qRT-PCR (Ct values) to create a three-tier range of viral load for the positive samples (right) compared to the clinically-annotated negative samples (class, red or grey). (bottom) The differentially expressed genes of SARS-CoV-2 positive patients compared to SARS-CoV-2 negative patients showed up-regulated (orange) genes as well as down-regulated (purple) genes. All differentially expressed genes with adjusted p-value < 0.001 and |log2 fold-change| > 1.5 (> 2.82 fold) are shown across different patient groups by viral level and presence of other viral pathogens. (b) Intersection heatmap of differentially expressed genes across different comparisons with genes in x-axis and comparisons in y-axis, with a core set of up-regulated genes (orange) distinct form the set of down-regulated genes (purple), compared to genes that are not significantly differently expressed (grey) in any comparison (Limma voom, q-value <0.01, |log2FC| >0.58).

Supp. Figure 10

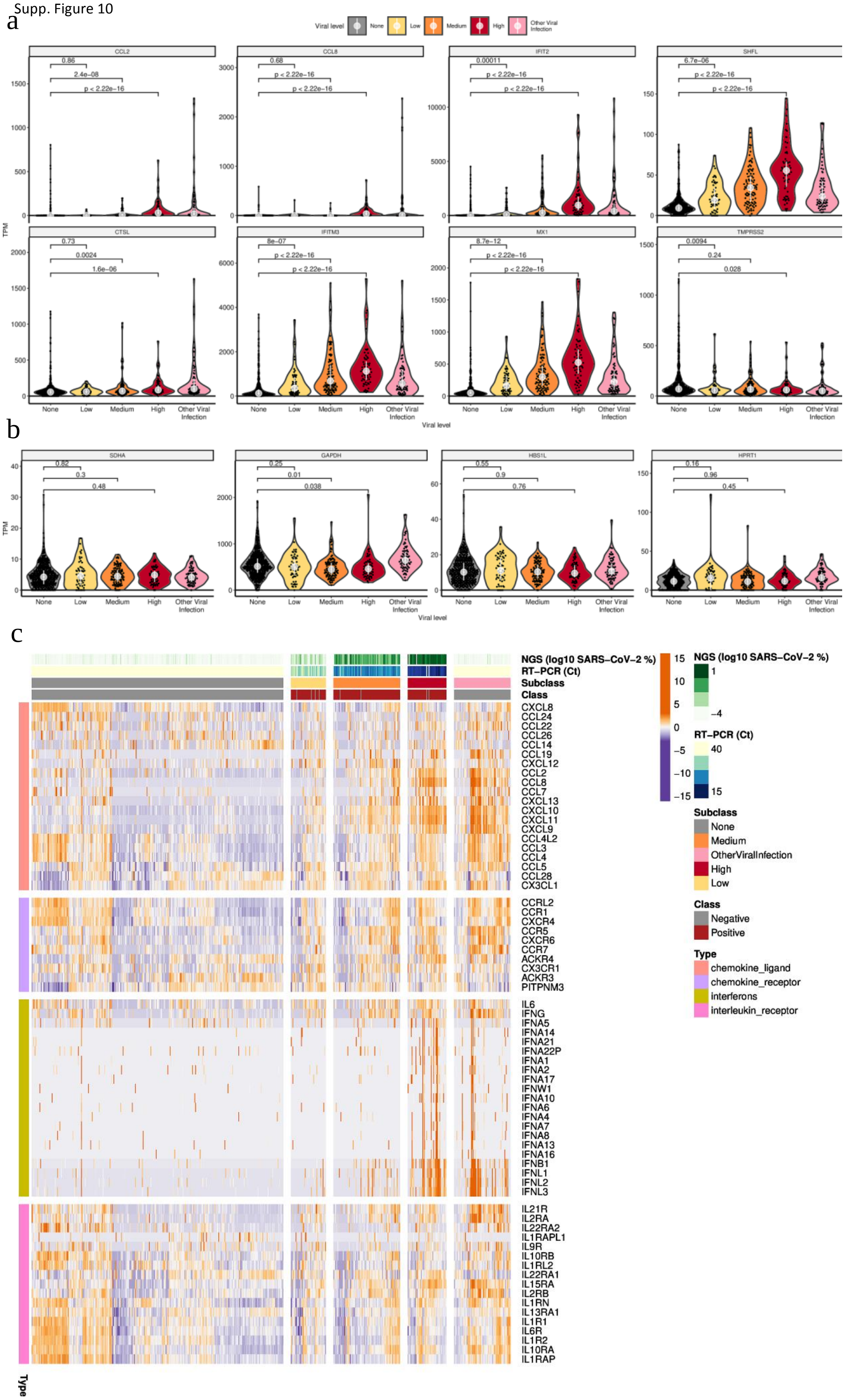

Supplementary Figure 10. Host gene expression relative to SARS-CoV-2 infection. Clinical samples were sequenced with RNA-seq and quantified to a set of genes for their expression levels. Samples with no virus (grey) were compared to those with low (yellow), medium (orange), and high (red) expression levels, based on qRT-PCR.p-values are calculated by Wilcoxon rank sum test and are not adjusted for multiple testing for a given gene. **(a)** Additional genes that were differentially expressed, or reported to be important for SARS-CoV-2 entry (adjusted p-values by negative binomial test in DESeq2) **(b)** Expression of housekeeping genes in different groups. **(c)** Cytokine and interferon profiles of the host transcriptome. Top rows: samples were quantified by a range of viral detection methods, including LAMP (QuantiFluor), RNA-seq (log10 SARS-CoV-2 % of reads), and qRT-PCR(Ct values) to create a three-tier range of viral load for the positive samples (right) compared to the clinically-annotated negative samples (class, red or grey). (bottom) The differentially expressed genes of SARS-CoV-2 positive patients compared to SARS-CoV-2 negative patients showed up-regulated (orange) genes as well as down-regulated (purple) genes. Heatmap is separated into Chemokine ligand, chemokine receptor, interferon and interleukin receptor profiles for the samples (x-axis) is plotted for each related gene (y-axis).

Supp. Figure 11

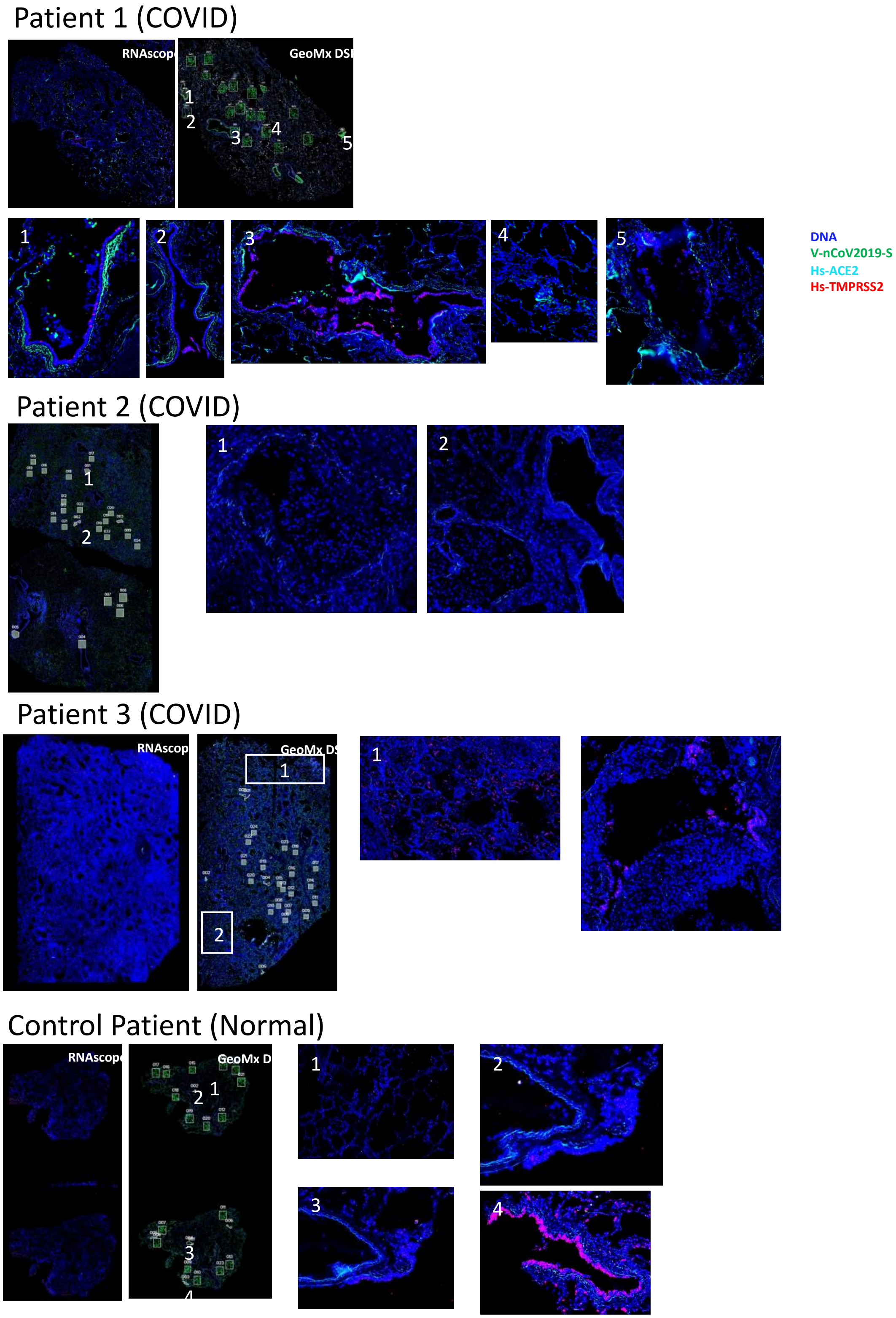

Supplementary Figure 11. Imaging from RNAscope and GeoMx DSP showing regions of gene expression measurement for COVID Patients #1, #2 and #3 along with close ups of numbered regions of interest (ROI).

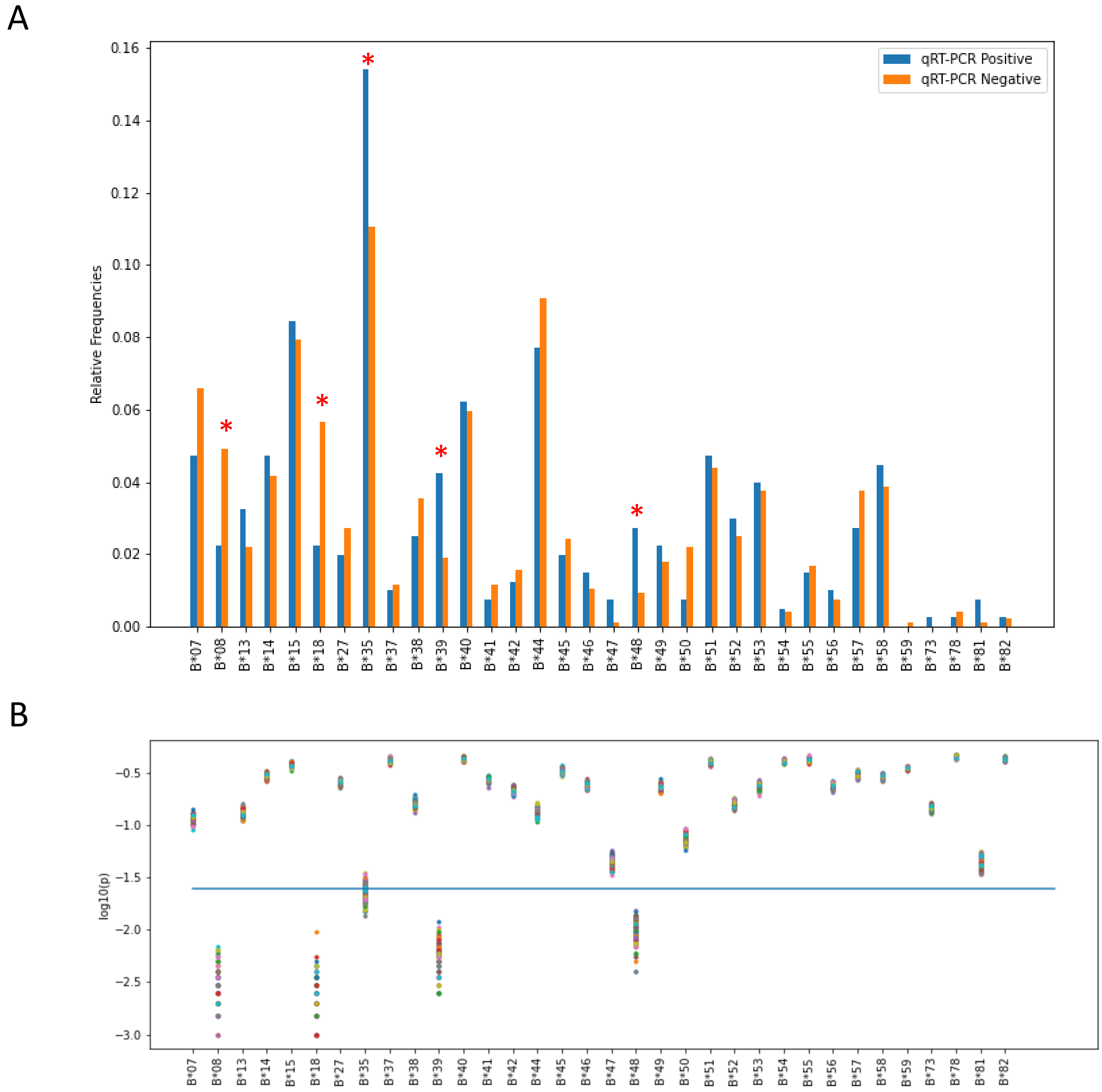

Supplementary Figure 12. Potential association of *HLA-B* type with COVID19. (A) Frequencies of *HLA-B* types among qRT-PCR-positive (blue) and -negative (orange) patients. Star (*) marks types nominally significant (by two-tailed Fisher exact test) first pass finding, prompting further testing by permutation. (B) Log-scale empirical probability (*p*) that label-permuted disparity in distribution of haplotype exceeds real case-control disparity, for 100 randomly seeded pools of 1000 permutations each.

A

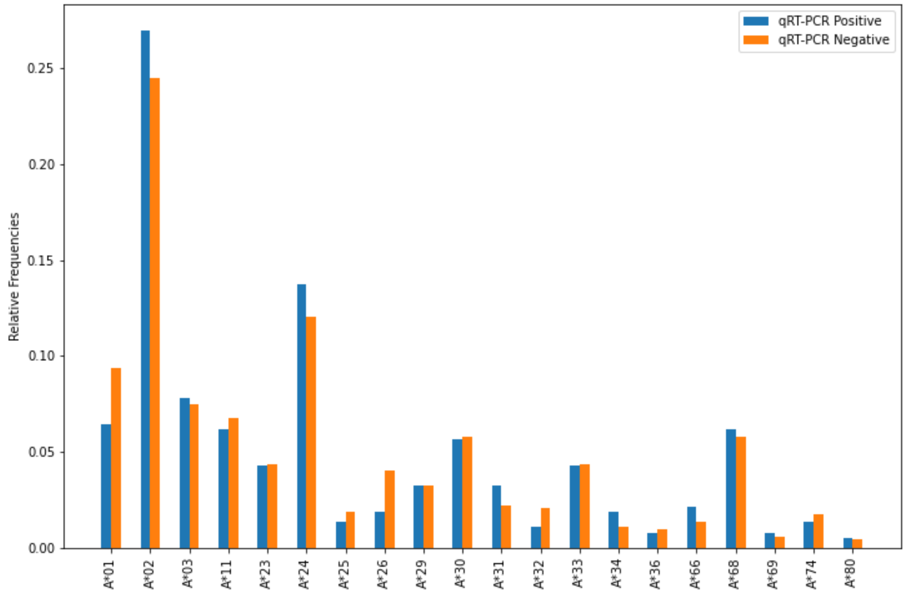

B

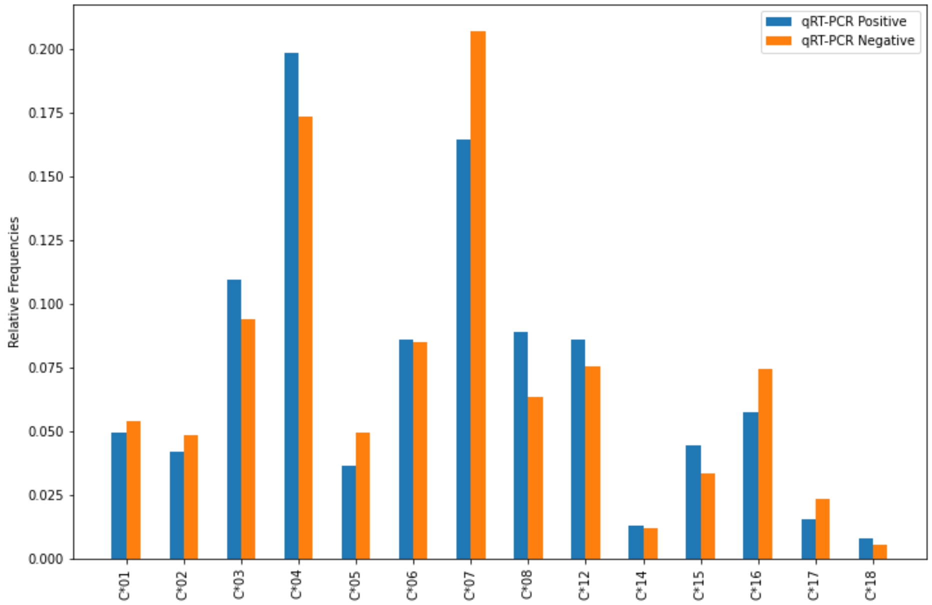

Supplementary Figure 13. Relative frequencies of other HLA types. (A) HLA-A frequencies; (B) HLA-C frequencies.
