# Supplementary material for: Shotgun transcriptome, spatial omics, and isothermal profiling of SARS-CoV-2 infection reveals unique host responses, viral diversification, and drug interactions
Source: Nat Commun. 2021 Mar 12;12:1660. doi: 10.1038/s41467-021-21361-7 (PMC7954844; doi:10.1038/s41467-021-21361-7)
Supplement: Supplementary file 3 — Description of Additional Supplementary Files [file 41467_2021_21361_MOESM3_ESM.docx]

Description of additional supplementary files:

**File name: Supplimentary_Data_1_TaxaClassification.xlsx**

**Description: Supplementary Table 1. Metatranscriptome Profiles of All Samples.**

**File name: Supplimentary_Data_2_PositiveNegativeTest.xlsx**

**Description: Supplementary Table 2. Taxonomic Mis-assignment Filter.**

Appended.

**File name: Supplimentary_Data_3_DEG_Pathways.xlsx**

**Description: Supplementary Table 3. Differentially Expressed Genes in SARS-CoV-2 +/- patients.** Appended.

**File name: Supplimentary_Data_4.xlsx**

**Description: Supplemental Table 4. Gene Ontology Pathways.**

Appended.

**File name: Supplimentary_Data_5_CrossReactivity.pdf**

**Description: Supplementary Table 5. SARS-CoV-2** **Rapid Colorimetric LAMP Detection Test: N Primers Specificity**

Appended.

**File name: Supplimentary_Data_6.xlsx**

**Description: Supplementary Table 6. Drug effect estimates from observational data**

Appended.

**File name: Supplimentary_Data_7.xlsx**

**Description: Supplementary Table 7: GISAID COVID-19 Acknowledgments**

Appended.
