# Supplementary material for: Shotgun transcriptome, spatial omics, and isothermal profiling of SARS-CoV-2 infection reveals unique host responses, viral diversification, and drug interactions
Source: Nat Commun. 2021 Mar 12;12:1660. doi: 10.1038/s41467-021-21361-7 (PMC7954844; doi:10.1038/s41467-021-21361-7)
Supplement: Supplementary file 8 — Supplementary Data 5 [file 41467_2021_21361_MOESM8_ESM.pdf]

Primer Cross Reactivity: N

			Primer / Primer Length					
			N_B3	N_BIP	N_F3	N_FIP	N_LB	N_LF
Organism Group	Organism Name	Organism ID	20 bp	40 bp	19 bp	41 bp	21 bp	25 bp
**Same genetic family**	SARS coronavirus 2	NC_045512.2	**100%**	45%-55%	**100%**	46%-54%	**100%**	**100%**
	SARS coronavirus	NC_004718	**85%**	43%-50%	**100%**	46%	**100%**	**88%**
	MERS coronavirus	NC_038294	0%	0%	0%	0%	0%	0%
	Human coronavirus NL63	NC_005831.2	80%	0%	0%	0%	0%	0%
	Human coronavirus 229E	NC_002645.1	0%	50%	0%	0%	0%	0%
	Human coronavirus HKU1	NC_006577.2	0%	0%	0%	0%	0%	0%
	Human coronavirus OC43 strain ATCC VR-759	NC_006213.1	0%	0%	0%	0%	0%	0%
**Other high priority organisms**	Candida albicans SC5314 chromosome 2 sequence	NC_032090.1		38%			**86%**	56%-64%
	Pneumocystis jirovecii RU7 chromosome Unknown supercont1.8	NW_017264782.1	**80%-85%**					64%
	Streptococcus pneumoniae NCTC7465, chromosome : 1	NZ_LN831051.1	**85%**			41%	62%	52%-64%
	Rothia mucilaginosa DY-18 DNA	NC_013715.1	80%					
	Chlamydia pneumoniae TW-183	NC_005043.1			79%			60%
	Candida albicans SC5314 chromosome 3 sequence	NC_032091.1					76%	52%-64%
	Pneumocystis jirovecii RU7 chromosome Unknown supercont1.14	NW_017264788.1						52%-76%
	Staphylococcus epidermidis ATCC 12228	NC_004461.1	65%-75%					52%-76%
	Candida albicans SC5314 chromosome 1 sequence	NC_032089.1	65%-75%	35%			62%	52%-60%
	Legionella pneumophila subsp. pascullei strain NCTC12273 , chromosome: 1	NZ_LR134380.1	65%-75%	35%				52%-72%
	Streptococcus pyogenes NCTC8198, chromosome : 1	NZ_LN831034.1	75%	35%			62%	52%-68%
	Bordetella pertussis 18323	NC_018518.1		55%-73%		37%-41%		72%
	Haemophilus influenzae NCTC8143, chromosome : 1	NZ_LN831035.1						52%-72%
	Mycoplasma pneumoniae FH chromosome	NZ_CP010546.1						60%-72%
	Pneumocystis jirovecii RU7 chromosome Unknown supercont1.15	NW_017264789.1						64%-72%
	Candida albicans SC5314 chromosome 4 sequence	NC_032092.1						52%-68%
	Candida albicans SC5314 chromosome R sequence	NC_032096.1						52%-68%
	Candida albicans SC5314 chromosome 5 sequence	NC_032093.1	65%					
	Candida albicans SC5314 chromosome 7 sequence	NC_032095.1	65%	35%				52%-56%
	Pneumocystis jirovecii RU7 chromosome Unknown supercont1.9	NW_017264783.1						64%
	Mycobacterium tuberculosis H37Rv	NC_000962.3				37%-59%		52%
	Candida albicans SC5314 chromosome 6 sequence	NC_032094.1						52%
	Pneumocystis jirovecii RU7 chromosome Unknown supercont1.10	NW_017264784.1						52%
	Pneumocystis jirovecii RU7 chromosome Unknown supercont1.12	NW_017264786.1						52%
	Pneumocystis jirovecii RU7 chromosome Unknown supercont1.17	NW_017264791.1						52%
	Pneumocystis jirovecii RU7 chromosome Unknown supercont1.3	NW_017264777.1						52%
	Pseudomonas aeruginosa PAO1	NC_002516.2		35%				

% Identity Color
- >80%
- <=80%

% Identity range (# identical bases/ # primer bases) is shown for each primer and organism. Darker font indicates % identity greater than 80% . Organisms with >= 50% identity primer hits are shown. This analysis is not intended to predict amplification. Near perfect homology across B3, F3, FIP and BIP is necessary to support successful amplification.
